# Cold Atmospheric Plasma in Wound Healing: Molecular Mechanism of Action

**DOI:** 10.3390/ijms27167130

**Published:** 2026-08-09

**Authors:** Dawid Bińkowski, Bogusław Antoszewski, Anna Kasielska-Trojan

**Affiliations:** 1 Faculty of Medicine, Medical University of Lodz, 90-419 Lodz, Poland; dawid.binkowski@stud.umed.lodz.pl; 2 Plastic, Reconstructive and Aesthetic Surgery Clinic, Institute of Surgery, Medical University of Lodz, 90-153 Lodz, Poland; b.antoszewski@wp.pl

**Keywords:** cold atmospheric plasma, plasma medicine, plasma, wound healing, regenerative medicine, reactive oxygen and nitrogen species

## Abstract

Cold atmospheric plasma (CAP) is a promising tool used in medicine due to its broad spectrum of biological activity and the possibility of safe application on living tissues without the risk of thermal damage. CAP is a partially ionised gas containing electrons, ions and reactive oxygen and nitrogen species (RONS); it also emits ultraviolet radiation and an electromagnetic field. The aim of this study is to discuss the mechanisms of action of cold atmospheric plasma and to assess its significance in supporting the skin healing process. Analysis of the available data indicates that CAP acts in multiple ways: it stimulates the proliferation and migration of keratinocytes and fibroblasts, modulates the inflammatory response, exhibits antimicrobial properties, and improves microcirculation and enhances angiogenesis. The results of studies to date suggest that cold atmospheric plasma may provide valuable support for therapies, particularly in the context of skin regeneration, wound healing and the treatment of inflammatory lesions. However, further research is needed on the standardisation of treatment parameters, long-term safety, and the precise definition of indications and contraindications depending on the type of device used.

## 1. Introduction

**Figure 1:**
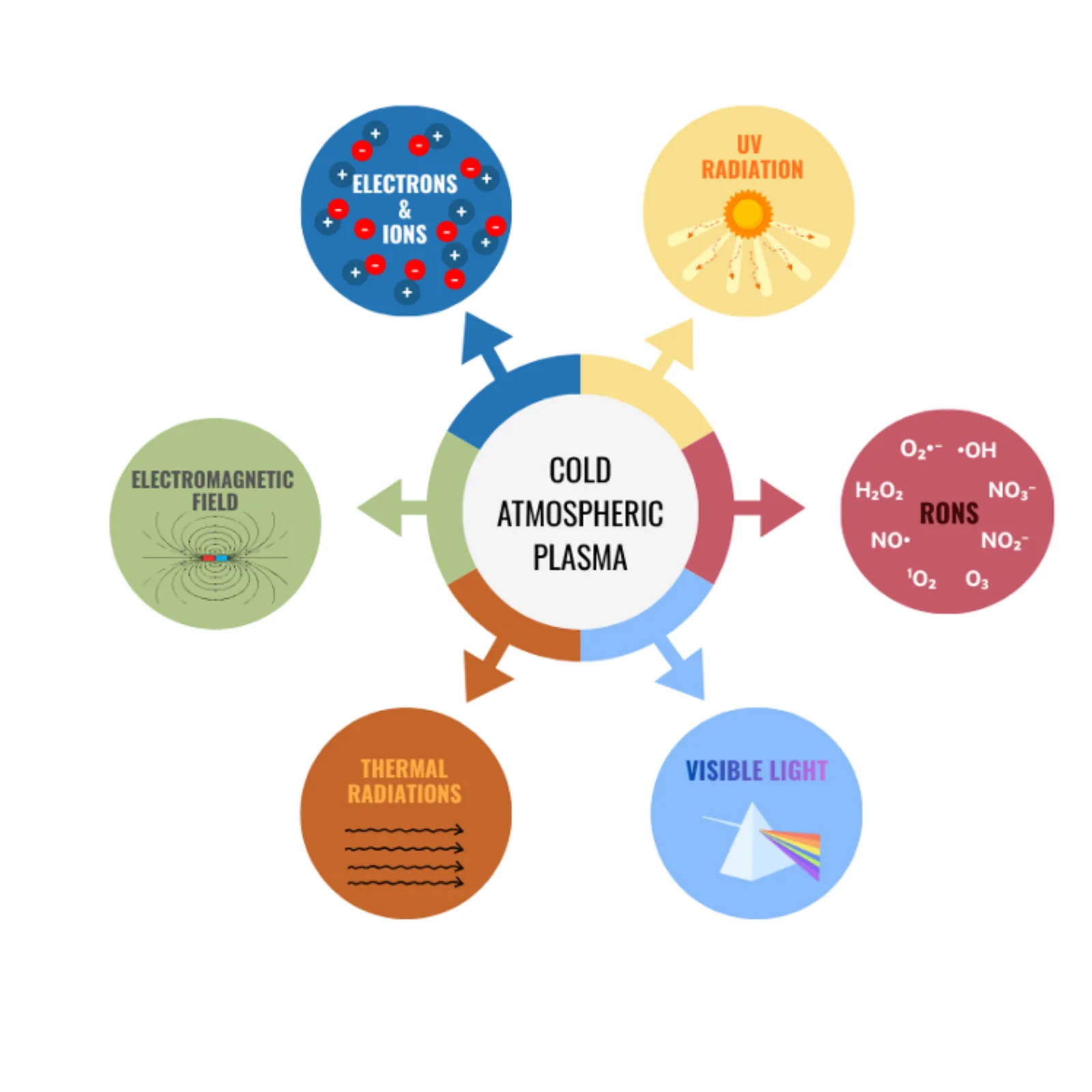
Composition of cold atmospheric plasma.

Plasma is commonly recognised as the fourth state of matter, alongside solids, liquids and gases. It is formed when a sufficient amount of energy is supplied to a gas, leading to its partial or complete ionisation [1]. From a physical point of view, for an ionised gas to be considered plasma, it must meet three basic conditions: (1) electrical quasi-neutrality, i.e., a similar number of positive and negative charges; (2) the ability of charged particles to shield electrostatic fields (Debye screening); and (3) the presence of a plasma frequency, describing the natural oscillations of particles leading to the restoration of electrical equilibrium [2,3]. Cold plasma consists of electrons, ions, neutral particles, reactive oxygen and nitrogen species; there is also ultraviolet radiation, an electromagnetic field, visible light and thermal radiation (Figure 1) [4].

This highly active environment is created by supplying energy to the gas, which leads to the ejection of electrons from the valence shells, i.e., ionisation. As a result of these processes, a mixture of reactive components is formed, and radiation is emitted [1,5]. The most significant property of plasma from a medical perspective is the fact that it contains a broad spectrum of reactive oxygen and nitrogen species (RONS), such as ozone, hydroxyl radicals, hydrogen peroxide and nitric oxide [6].

The key classification of plasma is based on its temperature, and more specifically on the system’s state of thermodynamic equilibrium [2,7]. Hot plasma, also known as thermal plasma, is characterised by the fact that all the components within it have the same temperature [8]. Cold plasma, on the other hand, also known as non-thermal plasma, does not exhibit such equilibrium. Free electrons have a significantly higher temperature than ions and neutral gas molecules. As a result, the plasma as a whole reaches a temperature close to room temperature, which allows it to be used safely directly on tissues [7,8]. In medicine, cold plasma generated at atmospheric pressure (CAP) has found the widest application. Carrier gases such as argon, helium, nitrogen or air are most commonly used for its production [9,10].

**Figure 2:**
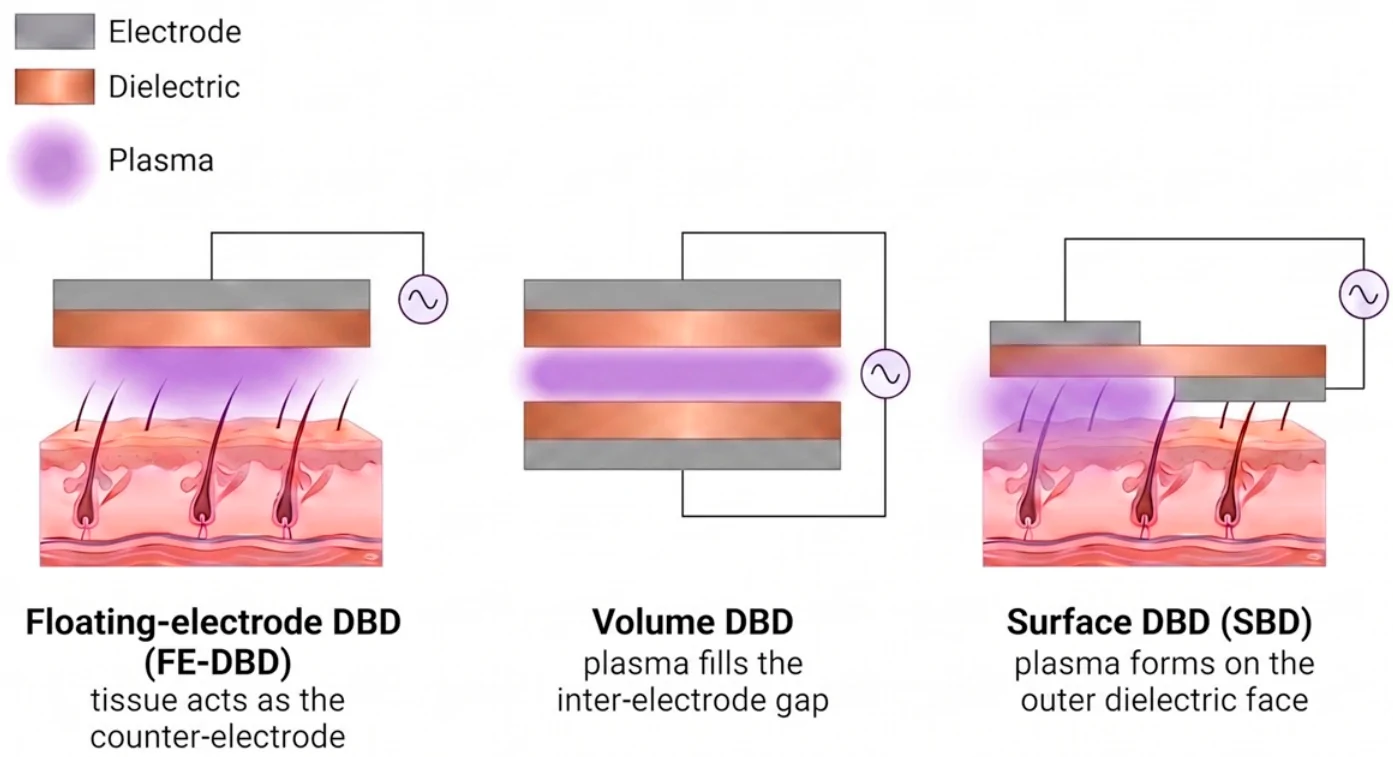
Dielectric barrier discharge. Created in BioRender. Bińkowski, D. (2026) https://BioRender.com/6bgf0eb. (accessed on 31 July 2026).

Cold atmospheric plasma can be obtained using a variety of techniques, differing in the mechanism of electrical discharge generation and the design of the apparatus. These processes are based on complex physical and chemical transformations. The most commonly used systems include dielectric barrier discharge (DBD) and atmospheric pressure plasma jet (APPJ). In a DBD, at least one electrode is covered by a dielectric layer (glass, ceramic or polymer) that limits the discharge current and keeps the plasma non-thermal, allowing safe treatment of living tissue [4,11]. In the volume configuration the plasma fills the gap between two electrodes; when the treated tissue itself acts as the counter-electrode—the floating-electrode DBD (FE-DBD)—the discharge is applied directly to the surface. In the surface-barrier configuration (SBD) both electrodes lie on the same dielectric and the plasma forms over its outer face, so the tissue is treated indirectly by reactive species diffusing toward it (Figure 2) [4,11]. Devices based on DBD include the PlasmaDerm® VU-2010 (CYNOGY GmbH, Duderstadt, Germany) and the PLASOMA® (Plasma-cure B.V., Nijmegen, The Netherlands) system, the latter applying the plasma directly to the wound with the tissue acting as the counter-electrode [12,13].

**Figure 3:**
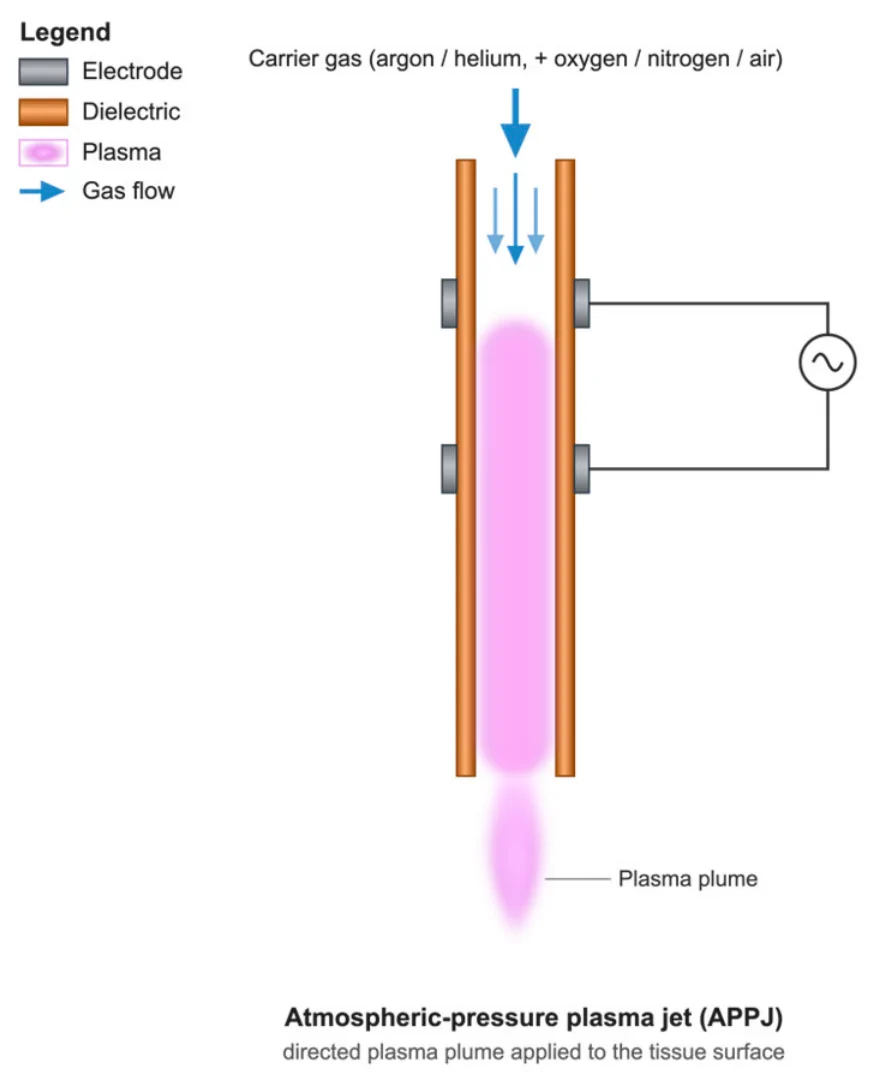
Atmospheric pressure plasma jet. Created in BioRender. Bińkowski, D. (2026) https://BioRender.com/ak082aj.

APPJ sources generate plasma within a capillary or nozzle by exciting a carrier gas—typically a noble gas such as argon or helium, sometimes with small admixtures of oxygen, nitrogen, or air—via alternating-current (AC), radiofrequency (RF), or pulsed excitation [4,11]. A gas flow then transports the plasma effluent out of the nozzle as a directed plasma plume that is applied to the tissue surface at a short working distance [4,11]. Because plasma cannot be stored, the discharge must be sustained during treatment, although in practice it is often operated in a pulsed or burst mode rather than continuously (Figure 3) [4,11]. Clinically certified examples include the argon-based kINPen® MED (Leibniz Institute for Plasma Science and Technology-INP Greifswald and neoplas med GmbH, Greifswald, Germany) and Adtec SteriPlas (Adtec Healthcare, Middlesex, UK), while the PlasmActionMed (ION BIOTEC S.L.Puertollano, Spain) device is air-based, illustrating that jets are not restricted to noble gases [11,14,15].

While DBD and APPJ remain the two principal source types, a broad and growing spectrum of alternative plasma source and delivery concepts is being developed. One example is the aerosol-based CAP-A approach implemented in the PLASMO®HEAL PRO system (WK-MedTec GmbH, Bückeburg, Germany) [16]. These methods allow for the production of low-temperature plasma, which can be used in various therapeutic procedures [4,17].

In clinical practice, cold plasma can be applied directly, based on the stimulation of selected tissues with ionised gas. The indirect method involves the use of plasma-activated fluids or media (e.g., PAW- plasma-activated water, PAS- plasma-activated solution), which contain reactive forms of oxygen and nitrogen [18]. The non-contact and painless nature of CAP means that this method is finding increasingly widespread application in medicine [19].

A key aspect of cold plasma’s action is that its properties and effects depend on the operating parameters of the generating device, in particular, the voltage and frequency of the pulses, as well as on environmental conditions, gas composition, distance from the tissue, and duration of exposure. These factors determine the amount of reactive oxygen and nitrogen species produced and the biological effect that will be induced in the tissues [1,6,8].

## 2. Mechanism of Plasma Action on Tissues

The mechanism of action of cold atmospheric plasma at the cellular level is based on direct action and the modulation of intracellular signalling pathways by its active physicochemical components [20]. The most important molecules generated by the plasma include: nitrites (NO_2_^−^), nitrates (NO_3_^−^), nitric oxide radical (NO•), hydroxyl radical (•OH), superoxide anion radical (O_2_•^−^), singlet oxygen (^1^O_2_), hydrogen peroxide (H_2_O_2_) and ozone (O_3_). Furthermore, CAP generates ultraviolet radiation and an electromagnetic field [8]. The effect of plasma is subject to the phenomenon of hormesis: short-term exposure and low concentrations of RONS stimulate metabolic pathways, inducing cell migration and proliferation, whereas high doses lead to programmed cell death, i.e., apoptosis [21,22]. In vitro studies have also demonstrated that CAP exhibits anticancer activity [23,24].

At the cell membrane level, highly reactive oxygen and nitrogen species, together with the electric field generated by CAP production, initiate lipid peroxidation, which causes temporary poration in the membrane structure. This leads to the destabilisation of the phospholipid bilayer, thereby facilitating the entry of key signalling molecules into the cytoplasm [25,26]. A similar process takes place in the stratum corneum, the outermost protective barrier of the epidermis. Cold atmospheric plasma locally increases the permeability of this layer and reduces its electrical resistance. As a result, small water-soluble substances that normally do not penetrate the skin can more easily cross the epidermal barrier. This may enable the effective, non-invasive transdermal delivery of active compounds [27].

### 2.1. Antimicrobial Effect

**Figure 4:**
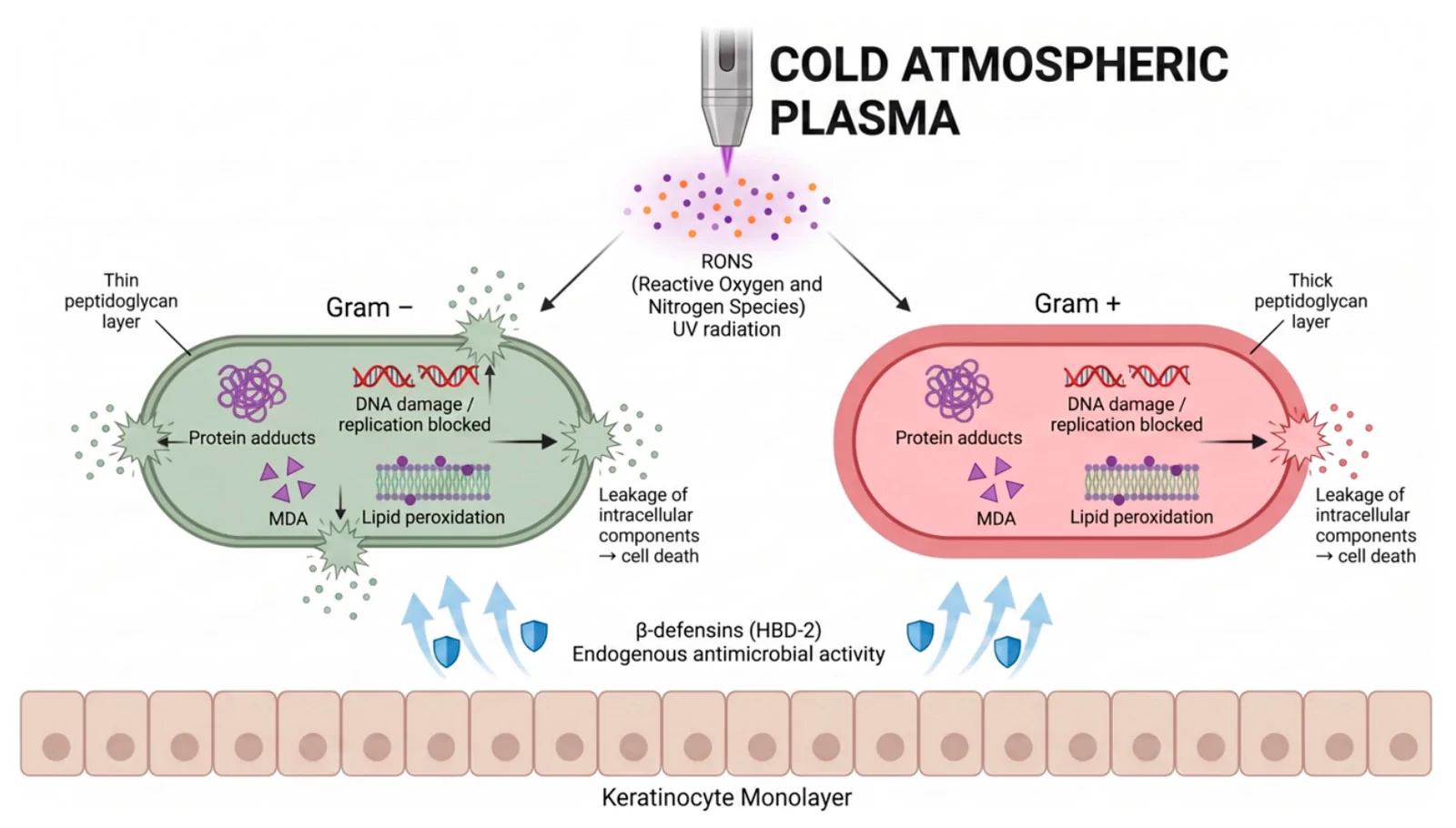
The effect of cold atmospheric plasma on bacterial cells. Created in BioRender. Bińkowski, D. (2026) https://BioRender.com/o6oj0kh. (accessed on 15 July 2026).

The antimicrobial effect of cold atmospheric plasma (CAP) results from the combined effect of reactive oxygen and nitrogen species and UV radiation on pathogenic microorganisms [28]. The multidirectional action of CAP on bacterial cells reduces the likelihood of them developing resistance, which is a highly desirable therapeutic effect [29]. The RONS generated during plasma treatment initiate membrane lipid peroxidation. According to Joshi et al., singlet oxygen and hydrogen peroxide are the principal species involved, and their action compromises membrane integrity, causing a loss of membrane potential and leakage of intracellular components. Lipid peroxidation also yields reactive aldehydes, including malondialdehyde (MDA), which can react with proteins and DNA, further exacerbating cellular damage [30]. In turn, Mai-Prochnow et al. found that Gram-negative bacteria were generally more sensitive to cold plasma than Gram-positive bacteria, and that this difference correlated with cell wall thickness: Gram-positive species, with their thick peptidoglycan layer, showed greater resistance, whereas the much thinner cell wall of Gram-negative species was associated with faster inactivation. The authors emphasise, however, that cell wall thickness is not the sole determinant. The correlation was statistically significant only after longer (10 min) treatment and did not hold within each Gram group at shorter exposure times, and additional factors—such as the outer membrane and pore-forming porins in Gram-negative bacteria, cell-surface appendages in Gram-positive bacteria, and the biofilm matrix—are also likely to influence susceptibility to CAP. Consistent with this, some Gram-negative species can be highly resistant despite a thin cell wall [31]. The action of RONS also involves inducing intracellular oxidative stress, which leads to oxidative damage to intracellular proteins and to nucleic acids such as DNA. As this damage accumulates, it exceeds the repair capacity of the cell, ultimately contributing to loss of viability and cell death [30,31]. Beyond its direct antibacterial action, Arndt et al. showed that CAP also stimulates keratinocytes to secrete β-defensins, in particular the human β-defensin 2 (HBD-2), which exhibits antimicrobial properties (Figure 4) [32]. The multifaceted effect of plasma on bacteria is an area of ongoing research and requires further investigation, particularly regarding the mechanisms by which it affects different types of microorganisms.

### 2.2. Cell Stimulation

Cold atmospheric plasma (CAP) stimulates key cell populations, such as keratinocytes, fibroblasts and vascular endothelial cells, to produce signalling molecules [33]. Studies have shown that CAP enhances keratinocyte migration, potentially supporting faster re-epithelialisation, while also increasing granulation tissue formation and promoting angiogenesis through autocrine and paracrine mechanisms [33,34,35]. These phenomena may facilitate the transition from the inflammatory phase to the repair and remodelling of the extracellular matrix in damaged tissue [36]. Multidirectional stimulation of proliferative processes significantly accelerates healing and allows the skin to rapidly restore its barrier function.

The effect of cold atmospheric plasma on keratinocytes depends on the duration of exposure and the plasma system used. Short-term exposure to CAP, lasting approximately 60 s, can accelerate keratinocyte migration and closure of the defect, as demonstrated in vitro studies using a dielectric barrier discharge operated in ambient air (~13.5 kV, 300 Hz) applied to keratinocytes covered by buffer [37]. Exposure lasting approximately 3 min stimulates the proliferation of basal layer keratinocytes, as evidenced by an increase in the number of Ki67-positive cells, shown for an argon plasma jet (kINPen MED, argon flow 5 L/min, 2–3 kV at 1 MHz) applied directly to ex vivo human skin biopsies [38]. Excessively long plasma exposure may have the opposite effect. Exposure of approximately 5 min has been shown to delay cell migration, enhance keratinocyte differentiation and increase apoptosis. These results demonstrate that the effect of CAP is time-dependent, although the optimal exposure differs between plasma devices and experimental models [37,38].

At the molecular level, cold atmospheric plasma, via reactive oxygen and nitrogen species, can activate important signaling pathways such as phosphoinositide 3-kinase/protein kinase B (PI3K/AKT) and mitogen-activated protein kinase/extracellular signal-regulated kinase (MAPK/ERK), which support cell migration and wound healing. Casein kinase 2 (CK2) also plays a significant role [39]. Concurrently, CAP may reduce the expression of E-cadherin, a protein responsible for intercellular junctions, and modulate integrin levels. These changes weaken cell adhesion and facilitate their migration towards the defect [40].

The effect of CAP on keratinocytes also occurs indirectly through the activation of fibroblasts located deeper within the dermis. Activation of the HIPPO/ Yes-associated protein (YAP) pathway induces the synthesis of connective tissue growth factor (CTGF) and cysteine-rich angiogenic inducer 61/cellular communication network factor 1 (Cyr61/CCN1) in fibroblasts, which, via paracrine signalling, enhance keratinocyte migration [41]. Under the influence of cold atmospheric plasma components, keratinocytes may exhibit increased expression and secretion of interleukin-8 (IL-8), which participates in the inflammatory response and promotes the recruitment of immune cells. Keratinocytes may also increase the production of factors associated with angiogenesis and wound healing, such as basic fibroblast growth factor (FGF-2), epidermal growth factor (EGF), endothelin-1, artemin, endocrine gland-derived vascular endothelial growth factor (EG-VEGF) and urokinase-type plasminogen activator (uPA) [32,33]. Furthermore, CAP may stimulate the production of β-defensins, i.e., peptides with bactericidal activity [32]. The production of these factors, together with increased proliferation of basal layer cells and enhanced keratinocyte migration, accelerates wound healing by stimulating the inflammatory response, angiogenesis and antimicrobial defence, whilst simultaneously leading to rapid re-epithelialisation, wound closure and restoration of the skin’s protective barrier.

Fibroblasts, i.e., cells located in the dermis, are also affected by the reactive oxygen and nitrogen species generated by plasma exposure. The effect of CAP on these cells is strictly dose dependent. Short-term exposure stimulates fibroblast proliferation, whereas prolonged stimulation may have a toxic effect on these cells, halting their division [21,30].

Cold atmospheric plasma triggers in fibroblasts a series of intracellular mechanisms associated with the wound healing process. Under the influence of CAP, fibroblasts are activated, which may promote their transformation into myofibroblasts. This is indicated by increased expression of smooth muscle α-actin (α-SMA) [20,42]. Activated fibroblasts and myofibroblasts participate in the synthesis and remodelling of the extracellular matrix, and may also be involved in processes related to wound contraction and closure [42,43]. Oxidative stress induced by CAP engages the HIPPO/YAP signalling axis in fibroblasts, and more specifically its transcriptional co-activator YAP. This leads to the secretion of CTGF and Cyr61/CCN1 proteins, which stimulates surrounding keratinocytes. The cells that make up the epidermis begin to migrate intensively towards the wound, which accelerates re-epithelialisation [41,44].

Reactive oxygen and nitrogen species present in cold atmospheric plasma may influence fibroblast activity and lead to changes in gene expression. Under the influence of CAP, fibroblasts increase the production of pro-inflammatory cytokines and chemokines, such as interleukin-6 (IL-6), IL-8 and monocyte chemoattractant protein-1 (MCP-1), which participate in the recruitment of immune cells, including neutrophils, monocytes and macrophages, to the site of injury. Additionally, stimulated cells may increase the expression and secretion of proangiogenic factors, such as vascular endothelial growth factor (VEGF) and angiogenin, which stimulate the formation of new blood vessels [20,33].

Activated fibroblasts and reactive chemical compounds present in plasma influence the composition of the extracellular matrix, which is essential for the mechanical strength of newly formed tissue. During such short-term exposure, CAP stimulates gene transcription and the synthesis of structural proteins in fibroblasts, particularly type I and type III collagen [21,42]. Plasma influences the secretion of matrix metalloproteinases and their tissue inhibitors [33]. The correct expression of these enzymes especially matrix metalloproteinase-1 (MMP-1) is crucial, as they participate in the degradation and remodelling of extracellular matrix components, including collagen, thereby creating space for newly synthesised fibres [45]. Additionally, this action paves the way for migrating keratinocytes [45]. Although CAP stimulates matrix production, it also acts as a regulator during the final stage of remodelling. It has been demonstrated that plasma has the potential to inhibit the transforming growth factor-beta/Mothers against decapentaplegic homolog (TGF-β/Smad) signalling pathway, which is associated with a reduction in the expression of TGF-β1, phosphorylated Smad2 (p-Smad2), phosphorylated Smad3 (p-Smad3), α-SMA and type I collagen. This association has been demonstrated in murine models [46]. This limits the excessive activity of fibroblasts and myofibroblasts. Consequently, wounds treated with plasma may have a smaller diameter and a lower risk of pathological scarring [46].

### 2.3. Improvement of Microcirculation and Stimulation of Angiogenesis

Angiogenesis, i.e., the process of forming new blood vessels from existing ones, is a significant mechanism from a dermatological perspective. It is responsible for proper blood supply, oxygenation and nourishment of tissues [33]. Cold atmospheric plasma (CAP) strongly stimulates this process at the cellular level in a direct manner, acting on cells located in the superficial layers. In turn, its indirect action is based on paracrine mechanisms, in which activated superficial cells release growth factors that stimulate deeper-lying endothelial cells to form new blood vessels, despite the absence of physical contact with the plasma stream [33]. One of the main causes of angiogenesis initiation under the influence of CAP are reactive nitrogen species (RNS) and reactive oxygen species (ROS). These molecules penetrate into tissues and act as crucial molecules that intracellular pathways [47].

Cold plasma supplies exogenous nitric oxide (NO) whilst simultaneously stimulating skin cells to increase the expression of endothelial nitric oxide synthase (eNOS), thereby enabling the production of endogenous nitric oxide within the damaged tissue. This relationship has been described, among other studies, in research on mouse models of burn wounds [47]. Increased NO concentration causes vasodilation, leading to improved microcirculation and tissue oxygenation, which significantly accelerates repair processes [48].

In keratinocytes forming the epidermis that have been exposed to plasma, increased activity of the hypoxia-inducible factor HIF-1α is observed; this factor participates in the regulation of angiogenesis and acts as one of the stimuli promoting the secretion of growth factors [49]. Under the influence of RONS, a cascade of proangiogenic factor secretion is triggered. In CAP treated keratinocytes, there is a significant increase in the expression of, among others, VEGF, FGF-2, artemin, endothelin-1 and IL-8. In contrast, in fibroblasts located in the dermis, the production of angiogenin, VEGF and other molecules involved in the regulation of angiogenesis, such as endostatin, is increased. These factors stimulate endothelial cells to divide and form tubular structures [33]. Furthermore, CAP activates the expression of fibroblast growth factor receptor 1 (FGFR1) and vascular endothelial growth factor receptor 1 (VEGFR1) in endothelial cells, making them more sensitive to the action of proangiogenic factors [33]. In summary, cold atmospheric plasma, by modulating angiogenesis processes, contributes to improved regeneration and wound healing.

### 2.4. Regulation of the Inflammatory Process

Cold atmospheric plasma (CAP) exhibits anti-inflammatory properties. Its effect involves the precise modulation of the immune response and oxidative stress [36]. A very important cellular mechanism is the activation of the nuclear factor erythroid 2-related factor 2 (NRF2)/Kelch-like ECH-associated protein 1 (KEAP1) antioxidant pathway. Under the influence of plasma, the NRF2 factor stimulates the production of detoxifying and antioxidant enzymes, which results in the neutralisation of excess reactive oxygen species and reduces the extent of damage associated with oxidative stress [36]. Furthermore, cold atmospheric plasma can stimulate macrophages to transition from the pro-inflammatory M1 phenotype towards a reparative, anti-inflammatory phenotype. This process reduces damage and promotes the release of factors that stimulate tissue repair [50].

## 3. Cold Atmospheric Plasma in the Treatment of Selected Types of Wounds

### 3.1. The Effect of Cold Atmospheric Plasma on the Healing of Diabetic Foot Ulcers

Diabetic foot ulcers (DFUs) are a severe complication of diabetes mellitus. Their healing is frequently impaired, and the wounds remain prone to prolonged inflammation and recurrent bacterial infection [51,52,53]. Cold atmospheric plasma (CAP) has been proposed as a therapeutic approach for accelerating the repair of chronic wounds [52,53]. In several randomized clinical trials, adding CAP to standard wound care improved clinical outcomes relative to controls [14,52,53].

The main clinical benefit of CAP in DFUs is faster wound closure and greater area reduction [14,52]. In a study using helium CAP, 77.3% of treated wounds shrank by at least 50% within three weeks, versus 36.4% under standard care [52]. A separate randomized trial with argon-based CAP found that treated wounds reached clinically meaningful area reductions of 10% and 20% significantly faster than the placebo group [14].

Wound healing is a dynamic process that proceeds through four continuous and overlapping phases: haemostasis, inflammation, proliferation, and remodelling [51]. Haemostasis stops the bleeding immediately after injury. During the inflammatory phase, immune cells are recruited to the wound to clear pathogens and cellular debris, a response coordinated by cytokines and growth factors. In the proliferative phase, new tissue is formed through angiogenesis, fibroblast activity, and re-epithelialisation, while the final remodelling phase reorganises the extracellular matrix and restores the skin’s barrier function [51,53]. Healing depends on the precise, timely progression through these phases, a coordination that is disrupted in diabetic foot ulcers [51,52].

The chronic nature of DFUs is associated with a prolonged inflammatory phase [52]. In normal wound healing, this phase is self-limiting, but in diabetes it does not resolve because a dysregulated pro-inflammatory response maintains elevated levels of pro-inflammatory cytokines in the wound bed [51]. CAP may act at this stage by modulating the local inflammatory response [51,52]. In DFUs, CAP exposure has been shown to lower several inflammatory mediators, including interleukin-1 (IL-1), IL-8, tumour necrosis factor-alpha (TNF-α), and interferon-gamma (IFN-γ). This reduction may allow the prolonged inflammatory phase to resolve and the wound to progress toward the proliferative phase [51]. CAP has also been linked to reduced wound exudate, with clinical improvement apparent by the third and sixth weeks of treatment [53].

Bacterial infections are an important factor that impair the healing of diabetic foot ulcers [51]. CAP possesses immediate antiseptic properties due to the generation of reactive oxygen and nitrogen species (RONS), which can disrupt bacterial function and reduce bacterial load. Clinical measurements demonstrate a significant reduction in bacterial load immediately following a CAP exposure session. Although wound bacteria may recolonize within ten hours, this immediate antiseptic effect, combined with cellular stimulation by CAP, is crucial for activating the wound healing process [52].

Across various trials, CAP therapy has proven to be safe and well-tolerated by patients with DFUs. Patients did not report severe therapy-related adverse events, thermal damage, or significant pain during the application. By effectively reducing wound size, alleviating chronic inflammation, and providing immediate antibacterial activity, CAP may represent a highly beneficial adjunctive therapy that accelerates the healing of diabetic foot ulcers [51,52,53].

### 3.2. The Effect of Cold Atmospheric Plasma on the Healing of Venous Leg Ulcers

Venous leg ulcers (VLUs) primarily result from the functional failure of venous valves in the lower limbs. This process leads to increased blood backflow and localized venous hypertension [54]. In the wound, an environment develops that promotes the persistence of chronic hypoxia, inflammation, and recurrent infections. Skin cells become less responsive to signals that would normally stimulate repair processes. As a result, the healing of wounds caused by venous insufficiency is impaired [12]. Together, these mechanisms make venous ulcers chronic and hard to heal.

Cold atmospheric plasma is a promising method for the treatment of venous ulcers because it acts through several different mechanisms [54]. CAP improves microcirculation. Immediately after plasma application, intense redness appears within the wound area, clinically referred to as the “flash effect.” This phenomenon is probably caused by local vasodilation [15]. In the study in which it was described, this effect was not shown to lead to clearly faster granulation under the treatment regimen used [15]. Cold atmospheric plasma has been shown to stimulate angiogenesis, modulate the inflammatory response, and influence the proliferation and migration of skin cells essential for re-epithelialisation. These processes are additionally supported by the beneficial acidification of the wound environment and the activation of integrin receptors on the cell surface [54].

Cold atmospheric plasma may also support healing through its antimicrobial and antiseptic effects. CAP therapy may represent an innovative treatment method because it may not only reduce the bacterial load in chronically infected wounds but also support tissue regeneration [54]. This is particularly relevant because plasma has been shown to act against pathogens such as antibiotic-resistant bacteria and biofilms [12]. Clinical observations also support this effect. In one reported case, Staphylococcus aureus was completely eliminated within two weeks of treatment initiation [54].

Available clinical data suggest that CAP may accelerate wound healing. It should be noted that in two controlled studies, the differences between the groups were not statistically significant, although the obtained results are promising [12,15]. In a randomized controlled trial involving 60 patients, the group treated with CAP showed a greater mean reduction in wound area after 17 weeks of therapy than the group receiving standard care (72.9% compared with 56.7%). Moreover, complete wound healing occurred more frequently in the CAP-treated group. Despite these promising results, the differences between the groups did not reach statistical significance (*p* > 0.05) [15]. The most important finding of this study was the rapid reduction in bacterial load after each CAP application. At weeks 0 and 4, the decrease in bacterial load was approximately 31–32% and was statistically significant (*p* < 0.05) [15].

In the second study, which included 46 patients, complete healing of venous ulcers was achieved in 61.5% of wounds treated with CAP twice a week and in 53.3% of wounds treated once a week. By comparison, in the standard care group, 25.0% of wounds healed completely. Despite the favourable results, these differences were not statistically significant. The authors note that this may have been due to the premature termination of the study and the small sample size [12]. In both CAP-treated groups, the wound area was much smaller than at the beginning of the study [12].

In another clinical study evaluating the effects of CAP on venous ulcers, it was shown that this therapy may influence wound surface area reduction and alleviate patient pain. Due to the limited sample size, these results did not reach statistical significance (*p* = 0.42). A strong but transient antibacterial effect was observed immediately after CAP application (*p* = 0.03), with subsequent recolonization after eight weeks [13]. The effectiveness of this method is also supported by a single severe case report. In a 65-year-old man, a venous ulcer with an area of 30.23 cm^2^ decreased to 10.62 cm^2^ within two weeks, and complete wound healing was achieved after four weeks. Additionally, elimination of Staphylococcus aureus was observed [54].

In summary, these findings suggest that CAP is a safe and non-invasive method supporting the treatment of chronic venous ulcers. Its effectiveness appears promising; however, it has not yet been clearly confirmed statistically. Therefore, larger clinical trials are needed to confirm this effect.

### 3.3. The Effect of Cold Atmospheric Plasma on the Treatment of Burn Wounds

Burn wounds are complex injuries characterized by three distinct zones of tissue damage. The central zone of coagulation undergoes irreversible necrosis, the zone of stasis involves reduced perfusion and ischemia, while the zone of hyperaemia features vasodilation and increased blood flow [55]. Deep burns, extending into the lower layers of the dermis, present a significant clinical challenge. These injuries are highly susceptible to infections, a dysregulated inflammatory response, and an elevated risk of hypertrophic scarring [55]. Second- and third-degree burns create a necrotic, protein-rich environment that facilitates the proliferation of multidrug-resistant pathogens. These lesions are particularly prone to colonization by Pseudomonas spp., notably P. aeruginosa, alongside Staphylococcus aureus, Klebsiella spp., Proteus spp., Enterococcus spp., and Escherichia coli [55,56]. Topical antimicrobials, such as silver-containing dressings, are routinely applied in the management of these conditions. It has been observed that they can exert cytotoxic effects on fibroblasts and keratinocytes, which delay the healing process [55]. Therefore, there is a need to develop new burn treatment methods that will reduce the risk of infection and support tissue regeneration.

Cold atmospheric plasma (CAP) may serve as a promising adjunctive therapy for burn wound management. This approach combines antimicrobial activity with the potential to support tissue regeneration [55]. The bactericidal effect of CAP is driven by the presence of RONS, which damage bacterial DNA while oxidizing and perforating the cell membranes of these microorganisms [55]. In vitro studies have demonstrated that plasma application leads to a local accumulation of nitrites, nitrates, and hydrogen peroxide, alongside environmental acidification. This phenomenon may contribute to reduced pathogen viability [56]. The antimicrobial action of cold atmospheric plasma is complemented by the previously described regenerative mechanisms. These involve the regulation of inflammation, enhanced skin cell proliferation, and the stimulation of angiogenesis. Specifically, CAP influences the inflammatory phase by governing the activity of macrophages and neutrophils, while also facilitating the proliferative phase through the modulation of fibroblast and keratinocyte functions [55].

A preclinical study using a porcine burn model, which closely resembles human skin, showed that Aceso cold plasma (ACP), a form of cold atmospheric plasma (CAP), does not impair wound healing. This was demonstrated in both deep partial-thickness and full-thickness burns. ACP treatment did not worsen parameters associated with hypertrophic scarring, such as dyschromia, elasticity, transepidermal water loss, and epidermal or dermal thickness. In full-thickness wounds, ACP was also associated with reduced bacterial bioburden, although the wounds were not deliberately infected [57].

Clinical evidence is also emerging. In a randomized controlled trial involving 40 pediatric patients with superficial partial-thickness and deep dermal burns, non-thermal atmospheric pressure plasma was used as an adjuvant therapy together with a biological dressing composed of cultured human keratinocytes (Epifast^®^) [58]. The results demonstrated a statistically significant reduction in the need for skin grafting within the CAP-treated cohort. The procedure was required in 10% of patients, compared to 40% of those receiving standard treatment with a biological dressing [58]. Moreover, the use of cold atmospheric plasma clearly reduced the incidence of skin infections. They occurred in 25% of patients treated with plasma, whereas in the conventional therapy group they affected nearly 38% of patients [58]. Although no statistically significant differences were observed in the length of hospital stay or analgesic consumption, plasma therapy was associated with a trend toward shorter hospitalizations and lower analgesic doses [58]. It is worth noting that the plasma-treated group avoided platelet concentrate transfusions entirely, whereas the control group required an average of seven units [58]. The observation suggests that CAP supports primary hemostatic processes during wound healing. According to the authors, this effect results from the ability of plasma to stimulate the production of growth factors and regulate inflammation [58].

A randomized clinical trial in adult patients with partial-thickness burns showed that adding cold atmospheric plasma (CAP) to modern dressings accelerates wound healing. Compared with the control group, patients treated with CAP achieved: faster wound-area reduction (*p* < 0.001), shorter time to complete epithelialization, with a median of 14 versus 21 days (*p* = 0.018), and lower pain intensity during dressing changes (*p* < 0.001). These results indicate that CAP may support tissue regeneration and, importantly, improve treatment comfort [59]. The characteristics and main outcomes of the clinical trials on the effect of CAP on wound healing discussed above are presented in Table 1.

Beyond clinical trials, CAP therapy is also employed in complex, real-world burn cases within routine medical practice. For example, one case report details a 40-year-old soldier with combat burns encompassing 23% of his total body surface area (TBSA). The injuries were colonized by multidrug-resistant pathogens, including Acinetobacter baumannii. As part of a treatment regime comprising the application of a biodegradable temporizing matrix (BTM) and micrografting, the patient additionally received daily large-area cold atmospheric plasma therapy [60]. During the course of management, the patient experienced an episode of Acinetobacter baumannii-induced sepsis, which was successfully resolved with systemic antibiotics [60]. As a result of the multimodal intervention, wound coverage reached approximately 90% following a procedure on day 53, and by day 70, healing was nearly complete [60]. Cold plasma was only one component of the treatment regimen, so its independent effect cannot be clearly determined. These results are promising but require confirmation in further clinical studies [60].

In summary, CAP appears to be a promising supportive therapy in burn wound management, particularly because of its antimicrobial activity and its effect on the wound healing process. Nevertheless, further studies are needed to establish treatment parameters and confirm the long-term safety of this method.

## 4. Limitations and Contraindications

At present, no clear contraindications against the use of cold atmospheric plasma have been identified and no formal guidelines specifying such contraindications exist. However, its use should take into account the patient’s condition, the nature of the lesions, and medical recommendations. It should be emphasised that studies utilising CAP therapy most frequently excluded patients with a pacemaker or an implantable cardioverter defibrillator, pregnant and breastfeeding women, as well as individuals with active cancer, undergoing immunosuppressive therapy, requiring revascularisation due to occlusive peripheral vascular disease, with renal failure requiring dialysis, systemic infection, diabetes mellitus and severe chronic diseases of internal organs [61,62]. Consequently, there is insufficient scientific evidence to allow for a definitive assessment of the safety of plasma use in these groups; therefore, this therapy is not currently recommended for these patients.

## 5. Conclusions and Prospects

Cold atmospheric plasma (CAP) is a promising therapeutic method that significantly improves and accelerates the healing process of both acute and chronic wounds. Its action is multifaceted. CAP has a strong antibacterial effect; furthermore, it stimulates regenerative processes, such as increased microcirculation and angiogenesis, and influences the proliferation and migration of skin cells, including fibroblasts and keratinocytes [2,33,48]. Plasma alters the expression of key genes associated with the healing process and also stimulates the synthesis of collagen essential for tissue regeneration. The widespread use of CAP is not limited solely to wound treatment. Plasma therapy is used in many fields of modern medicine, including dermatology, oncology, surgery, dentistry and cosmetology [2,4,5]. This is due to the fact that CAP is a rich source of reactive oxygen species (ROS) and reactive nitrogen species (RNS), which have a wide range of effects on many cells [6,8].

Despite its enormous potential, cold plasma therapy still faces many challenges. One of the greatest is the lack of fully standardised protocols and the need to establish a precise unit dose that is easily measurable and controllable. Short-term exposure stimulates tissue regeneration, whereas too high an intensity or too long a duration of treatment may lead to necrosis or the cessation of healing processes [6,8]. This challenge is compounded by the diversity of devices in use, which rely on different plasma generation principles and differ in their operating parameters, resulting in a different composition of reactive components and limited comparability of the results obtained [4]. The development of optimal guidelines for specific devices is therefore essential for safe, routine use. It is therefore crucial to further explore the mechanisms of CAP action to enable the safe, effective and widespread use of this technology in medicine.

## Figures and Tables

**Table 1:** Summary of clinical trials on the effect of cold atmospheric plasma (CAP) on wound healing.

Author (Year)	Wound Type	Study Design	No. of Patients	Intervention	CAP Technical Parameters	Results—Wound Healing	Results—Bacterial Load
Amini MR et al. (2020) [45]	DFU	RCT	44 (22 SOC vs. 22 SOC + CAP)	5 min, 3×/week for 3 weeks	Helium plasma; voltage 10 kV, frequency 6 kHz, flow rate 2 L/min	Levels of pro-inflammatory cytokines (IL-1, IL-8, INF-γ, TNF-α) after CAP were significantly lower than in the control group (*p* = 0.001). Conclusion: Resolution of the inflammatory phase and acceleration of wound healing.	Reduction in bacterial count after each plasma session (*p* < 0.0001). Conclusion: Support of wound healing through antibacterial action.
Mirpour S et al. (2020) [46]	DFU	RCT	44 (22 SOC vs. 22 SOC + CAP)	5 min, 3×/week for 3 weeks	Helium plasma; voltage 10 kV, frequency 6 kHz, flow rate 2 L/min	Greater reduction in wound surface area in the CAP-treated group (*p* = 0.02); proportion of wounds reduced by half after 3 weeks: 77.3% (plasma) vs. 36.4% (control)(*p* = 0.006). Conclusion: Acceleration of wound healing.	Reduction in bacterial count immediately after CAP application (*p* < 0.05); recolonization after about 10 h. Conclusion: Strong but short-lived antiseptic effect.
Stratmann B et al. (2020) [48]	DFU	RCT	43 patients/62 wounds (31 CAP vs. 31 placebo)	8 applications over 14 days, 30 s/cm^2^	Argon plasma (kINPen Med)	CAP showed greater wound surface reduction (remaining area: 30.5% vs. 55.2% in placebo) (*p* = 0.03); significantly earlier wound reduction of 10% (*p* = 0.01) and 20% (*p* = 0.001). Conclusion: Acceleration of wound healing.	Reduction in bacterial count similar in both groups (53% vs. 50%) (*p* = 0.59). Conclusion: No significant effect of plasma on bacterial load.
Samsavar S et al. (2021) [47]	DFU	RCT	20 (10 SOC vs. 10 SOC + CAP)	2×/week for 6 weeks, 1 min/cm^2^	Helium plasma; voltage 4.5 kV, frequency 22 kHz; flow rate 6 L/min	Significant decrease in ulcer size at the end of the treatment period (*p* = 0.007); wound grading improved significantly in the sixth week (*p* = 0.019); significant reduction in wound exudate in the third (*p* = 0.039) and sixth weeks (*p* = 0.012). Conclusion: Plasma treatment accelerated the wound healing process.	Only the type of bacteria was assessed. Conclusion: No quantitative data on the antibacterial effect.
Rodríguez-Ferreyra P et al. (2025) [56]	BW	RCT	40 (20 SOC + Epifast^®^ vs. 20 SOC + CAP + Epifast^®^)	daily, ~1 min/10 cm^2^	Helium plasma; frequency 13.56 MHz, power 18–20 W; flow rate 0.5 L/min	Significant reduction in skin grafts in the CAP group: 10% vs. 40% in the control group (*p* = 0.02); non-significant trend toward shorter hospital stay (9.85 days CAP vs. 11.65 days control) (*p* = 0.38) and lower analgesic consumption (13.01 doses CAP vs. 21.15 doses control) (*p* = 0.09). Conclusion: Accelerated healing and reduced surgical morbidity.	Lower infection rate (25% after CAP vs. 37.95%) (*p* < 0.05). Conclusion: Reduction in wound infection risk.
A CJ et al. (2025) [57]	BW	RCT	36 (18 CAP + dressing vs. 18 dressing)	3×/week, 5–8 min, 10–15 mm distance from the wound bed, applied until healed (maximum follow-up of 28 days)	Handheld plasma device	Faster wound area reduction than in the control group (*p* < 0.001); faster complete epithelialization (median 14 vs. 21 days) (*p* = 0.018); less pain during dressing changes (*p* < 0.001). Conclusion: Acceleration of healing and reduction in pain.	No data on the antibacterial effect.
Bakker O et al. (2025) [50]	VLU	RCT	46 (15 SOC vs. 17 SOC + CAP 1×/week vs. 14 SOC + CAP 2×/week)	2 min, 1× or 2×/week for 12 weeks	Direct-CAP (PLASOMA)	More wounds healed at 12 weeks: 25% (control) vs. 53.3% (1×/week) vs. 61.5% (2×/week)- not statistically significant (*p* = 0.16 for 1×/week, *p* = 0.08 for 2×/week). Significant wound area reduction compared to baseline within both plasma groups (*p* < 0.001), but not in the control group (*p* = 0.11). Conclusion: Despite the lack of statistical significance, plasma treatment in patients with venous ulcers substantially increased the proportion of healed wounds.	No data on the antibacterial effect.
Henares A et al. (2026) [51]	VLU	RCT	60 (30 SOC vs. 30 SOC + CAP)	60 s/cm^2^, 2×/week for 10 weeks	Air-based cold atmospheric plasma (PlasmAction Med)	Greater wound area reduction (−72.9% vs. −56.7%; *p* = 0.30) and higher complete healing rate (42.9% vs. 30.4%; *p* = 0.361) at week 17, not statistically significant. Conclusion: Despite the lack of statistical significance, a clinically favorable wound-healing trend.	Significant reduction in bacterial load immediately after plasma application at week 0 (*p* = 0.016) and week 4 (*p* = 0.014), disappearing at week 9 (*p* = 0.736). Conclusion: Rapid but transient antimicrobial effect.
Brehmer F et al. (2015) [52]	VLU	RCT	14 (7 SOC vs. 7 SOC + CAP)	3×/week for 8 weeks, 45 s/cm^2^	DBD (PlasmaDerm VU-2010)	Wound size reduction >50% in 4/7 patients (SOC + CAP) vs. 5/7 (SOC). Greater absolute mean size reduction in the plasma group (−5.3 cm^2^ vs. −3.4 cm^2^), difference not statistically significant (*p* = 0.42); less pain was noted in the plasma group. Conclusion: Favorable clinical trend in wound healing and pain reduction, lacking statistical significance due to the very small sample size.	Significant reduction in bacterial count immediately after plasma application (*p* = 0.03); recolonization after 8 weeks. Conclusion: Strong but transient antibacterial effect.

CAP—cold atmospheric plasma; SOC—standard care; RCT—randomized controlled trial; DFU—diabetic foot ulcer; BW—burn wound; VLU—venous leg ulcer; DBD—dielectric barrier discharge. Entries [51] and [52] present different endpoints of the same cohort of 44 patients. Electrical and optical characterization of the plasma source–Supplementary Material.

## Data Availability

No new data were created or analyzed in this study. Data sharing is not applicable to this article.

## Supplementary Materials

The following supporting information can be downloaded at: https://www.mdpi.com/article/10.3390/ijms27167130/s1, Electrical and optical characterization of the plasma source included in Table 1
