# Supplementary material for: Cold Atmospheric Plasma in Wound Healing: Molecular Mechanism of Action
Source: Int J Mol Sci. 2026 Aug 9;27(16):7130. doi: 10.3390/ijms27167130 (PMC13513933; doi:10.3390/ijms27167130)
Supplement: Supplementary file 1 [file ijms-27-07130-s001.zip › ijms-4458356-supplementary.pdf]

Amini MR et al. (2020) [51] and Mirpour S et al. (2020) [52]

Helium plasma; voltage 10 kV, frequency 6 kHz, flow rate 2 L/min

Source geometry: Pyrex nozzle (ID 2 mm, OD 4 mm), 10 mm-wide copper powered electrode wrapped around the tube, nozzle-to-wound distance ~10 mm.

Electrical characterisation: DC-pulsed HV supply, 10 kV, 6 kHz, duty cycle 20%, supply input power 30 W. Short pulse on-time to limit heating. Discharge current, transferred charge and dissipated plasma power not reported.

Spectroscopic temperature: plume gas temperature ~32 ± 2 °C (rotational-temperature measurement, N_2 second positive system).

Optical characterisation (OES): AvaSpec-ULS 2048 spectrometer, 200-1100 nm, 0.5 nm resolution. Identified emission: RNS bands 300-500 nm, He lines (~500-700 nm), atomic O at 777 nm, OH at 310 nm, NO at 297 nm; UV mainly in 300-400 nm (limited DNA-damage potential).

Reactive species: O, OH, O_2^-, NO, H_2O_2 (ROS/RNS) and excited He states.

Not reported: electron/species number densities, spatial distribution of the plasma column, fast imaging.

Stratmann B et al. (2020) [14]

Argon plasma jet (kINPen Med)

The clinical study by Stratmann et al. [14] does not itself report the electrical or optical diagnostic parameters of the plasma source; the characterisation summarised below was compiled from the available literature describing this device - Bekeschus, S.; Schmidt, A.; Weltmann, K.-D.; von Woedtke, T. The Plasma Jet kINPen-A Powerful Tool for Wound Healing. Clin. Plasma Med. **2016**, 4, 19-28. https://doi.org/10.1016/j.cpme.2016.01.001

Source geometry: central rod electrode shielded by a dielectric quartz capillary + grounded ring electrode; effluent length 9-11 mm, diameter ~1 mm. Recommended working distance 8-10 mm.

Electrical characterisation: sinusoidal voltage 2-6 kV(pp), HF frequency 1 MHz, pulsed at 2.5 kHz (plasma on/off 1:1). Power < 3.5 W (hand-held unit), < 8 W (whole system). Discharge current, transferred charge and dissipated energy not reported.

Feed gas: argon 5 ± 1 L/min.

Spectroscopic temperature: 35-38 °C at the recommended working distance (thermal-camera / non-thermal operation).

Optical characterisation (OES): emission mainly in UV-A and UV-B, very low UV-C. Effective UV irradiance at working distance UVA 5-15 µW/cm², UVB 5-15 µW/cm² (60 s free-jet effective dose 1.05 J/m²). Fast imaging: bullet-like plasma propagation reported.

Reactive species: ROS/RNS with H_2O_2 dominating the liquid phase. On contact with ambient air H_2O_2, O_3, •OH, NO generated.

Not reported: electron/species number densities, spatial mapping of species distribution.

Samsavar S et al. (2021) [53]

Helium plasma; voltage 4.5 kV, frequency 22 kHz; flow rate 6 L/min

Source geometry: two 20 mm copper disks (HV and grounded electrode) with seven holes, 20 mm apart, holding seven 4 mm quartz tubes; ABS spacer/body.

Electrical characterisation: AC supply, 4.5 kV peak-to-peak, 22 kHz. Electrical behaviour measured with HV probe (Tektronix P6015A, 1:1000), current probe (TCP-202) and oscilloscope (Tektronix DPO3012) - instrumentation reported, but discharge current, transferred charge, power and dissipated energy values not given.

Optical characterisation (OES): performed with an Avantes UL-3648 spectrometer to obtain relative intensities of reactive species (ROS/RNS) - method reported, but no spectrum, identified lines/bands or quantitative species data given.

Thermal characterisation: gas temperature monitored with a non-contact camera (FLIR E4) under equilibrium conditions - no numerical temperature reported.

Reactive species: ROS/RNS (qualitative).

Not reported: numerical OES lines/species intensities, electrical discharge values, gas temperature value, electron/species densities, spatial distribution, fast imaging.

Rodríguez-Ferreyra P et al. (2025) [58]

Helium plasma; frequency 13.56 MHz, power 18-20 W; flow rate 0.5 L/min

Source geometry: purpose-built reactor with an 8 mm outlet nozzle (optimised by simulation for wide, uniform coverage). Anode-dielectric-cathode jet configuration.

Electrical characterisation: radio-frequency source 13.56 MHz via matching box. Power 18-20 W. Irradiance 0.420-0.560 W/cm² (mean 0.4659 W/cm²), below the ICNIRP 4 W/cm² safety threshold. (Discharge current, transferred charge and dissipated energy not reported.)

Gas temperature: reactor outlet kept < 27 °C (thermal control, no thermal damage).

Optical characterisation (OES): full emission spectrum recorded, 200-500 nm. NO bands (~200-260 nm), OH, N_2 and N_2^+ (~300-430 nm)

Reactive species: ROS -•OH, singlet oxygen, H_2O_2 (by absorbance). RNS - NO (γ), NO_2, N_2^+, peroxynitrite ($ONOO^-$) noted.

Not reported: electrical discharge values (current/charge/energy), electron/species number densities, spatial distribution mapping, fast imaging

A CJ et al. (2025) [59]

No source-characterisation data for this device could be found in the available literature.

Bakker O et al. (2025) [12]

Direct-CAP, DBD (PLASOMA)

Some physical characterisation of this plasma source exists in the literature. However, as it refers to an earlier prototype rather than the certified device used here, these values were not included as parameters of the present trial.

Henares A et al. (2026) [15]

Air-based cold at-mospheric plasma jet (PlasmAction Med)

The clinical study by Henares et al. [15] does not itself report the electrical or optical diagnostic parameters of the plasma source; these were compiled from the available literature describing this device - Cortázar, O.D.; Megía-Macías, A.; Hontanilla, B.; Cortázar-Gallicchio, H. Demonstration of the High Efficiency of an Air Plasma Jet Combining Electric Field and RONS in the Treatment of Chronic Wounds. IEEE Trans. Radiat. Plasma Med. Sci. **2025**, 9, 680-688. https://doi.org/10.1109/TRPMS.2024.3486975

Source geometry: concentric-electrode jet. Frustoconical powered electrode and flat ground electrode separated by a ~2 mm gap, air flow 10 L/min, 2 mm outlet orifice, stainless-steel removable sleeve. Skin-surface distance in clinical use ~8-16 mm.

Electrical characterisation: RF HV supply, 15 kV RMS at 30 kHz. Discharge running values 2.5 kV RMS and 5 mA RMS, RMS power ~15 W.

Spectroscopic/gas temperature: not spectroscopically derived. IR camera gives skin-surface temperature <40 °C (low heat transfer, out-of-equilibrium jet).

Optical characterisation (OES): UV-VIS emission spectrum (200-400 nm) reported. Identified: NO bands (200-280 nm), OH (280-310 nm), N_2 (310-380 nm), overlap of $O/O^+/N_2^+$ (380-400 nm), Cu electrode lines (324.8, 327.4 nm). UV irradiance 0.074 W/m^2 (radiometer), max UV exposure ~6.76 min.

Reactive species: RONS - NO, OH, N_2, plus $O/O^+/N_2^+$ (from OES); electric field emphasised as co-mechanism.

Electric field: measured in the jet (electro-optical probe), peak 10-30 V/mm over 8-20 mm - reported as first such measurement in an air jet.

Not reported: electron/species number densities, fast imaging.

Brehmer F et al. (2015) [13]

DBD (PlasmaDerm VU-2010)

Only the parameters reported for the specific device used here are given, as this plasma source exists in several versions that may differ, values from other configurations were not included.

Source geometry: hand-held DBD applicator, dielectric-covered HV electrode. Skin acts as counter electrode. Contact-free application at fixed <1 mm distance via a sterile exchangeable spacer. Treats ~1 cm^2 per application (moved across the ulcer). Line voltage 230 VAC, overall device 294 × 185 × 76 mm, 2.1 kg.

Electrical characterisation: non-equilibrium, weakly ionised DBD driven by alternating voltage pulses, amplitude >10 kV, power density 120 mW/cm^2. Dielectric limits current to prevent arcing. Mains/line supply: 230 VAC, 50 Hz, max power consumption 8 VA (device power-supply specs). Drive/discharge frequency of the DBD not reported. Discharge current, transferred charge and dissipated plasma power not reported.

Gas temperature: not reported. No spectroscopic temperature.

Optical characterisation (OES): not reported.

Reactive species: not measured.

Not reported: discharge current, charge, dissipated power/energy, OES/spectra, spectroscopic gas temperature, electron and species number densities, fast imaging, spatial discharge distribution.
